# Differential effects of knee flexion on Caton–Deschamps, patellotrochlear and Insall–Salvati indices: A dynamic magnetic resonance imaging—controlled study

**DOI:** 10.1002/jeo2.70727

**Published:** 2026-05-18

**Authors:** Oliver Swietek, Christian Arras, Alexander Korthaus, Matthias Krause, Karl‐Heinz Frosch, Jannik Frings

**Affiliations:** ^1^ Department of Trauma and Orthopaedic Surgery University Medical Center Hamburg‐Eppendorf Hamburg Germany; ^2^ Department of Trauma, Hand and Reconstructive Surgery, Klinikum Dortmund University of Witten/Herdecke Dortmund Germany; ^3^ Department of Trauma Surgery, Orthopaedics and Sports Traumatology BG Klinikum Hamburg Hamburg Germany; ^4^ Department of Orthopaedic and Trauma Surgery University Medical Center Schleswig‐Holstein, Campus Luebeck Luebeck Germany

**Keywords:** dynamic MRI, patellar height, patella alta, patellar instability, patellotrochlear Index

## Abstract

**Purpose:**

To evaluate the influence of knee flexion on patellar height measurements during active muscle movement using dynamic magnetic resonance imaging (MRI).

**Methods:**

Dynamic magnetic resonance (MR) images from 48 healthy participants were analyzed. Images were acquired over 30 s using a 3‐Tesla MRI unit during active knee motion between 40° of flexion and full extension. Sagittal images were used to determine the femorotibial flexion angle (FTA), and patellar height was assessed at increasing flexion angles (5°–10° increments) using the Caton–Deschamps index (CDI), Insall–Salvati ratio (ISR) and patellotrochlear index (PTI). Measurements were performed independently by two raters on two separate occasions. Statistical analysis was performed using mixed‐effects models and regression analyses.

**Results:**

Eighty knees from 48 individuals (24 males, 24 females; age 26.7 ± 5.0 years; height 176 ± 9.5 cm) were included. Within a mean active range of motion from 4.3° ± 7.8° to 38.5° ± 10.4°, patellar height changed differentially across all parameters. CDI (mean in extension 1.19 ± 0.16, 95% confidence interval [CI]: 1.10–1.19) decreased by 0.004 per degree of flexion (*p* < 0.001), ISR (mean in extension 1.19 ± 0.16, 95% CI: 1.15–1.24) decreased by 0.001 per degree of flexion (*p* < 0.001), and PTI (mean in extension 0.26 ± 0.20) increased by 0.02 per degree of flexion (*p* < 0.001). Inter‐ and intra‐observer reliability were excellent for CDI (ICC 0.91/0.86) and PTI (ICC 0.97/0.98), and good for ISR (ICC 0.76/0.75).

**Conclusion:**

Patellar height measurements strongly depend on knee position, particularly at low flexion angles. CDI and PTI demonstrated significant changes, whereas changes in ISR were minimal. Patellar height should therefore be interpreted with careful consideration of knee position and the specific index used.

**Level of Evidence:**

Level III, diagnostic study.

## Graphical abstract

Patellar height strongly depends on knee position at low flexion angles, significant changes in Caton–Deschamps index and patellotrochlear index, but minimal changes in ISR, requiring careful interpretation of measurements.

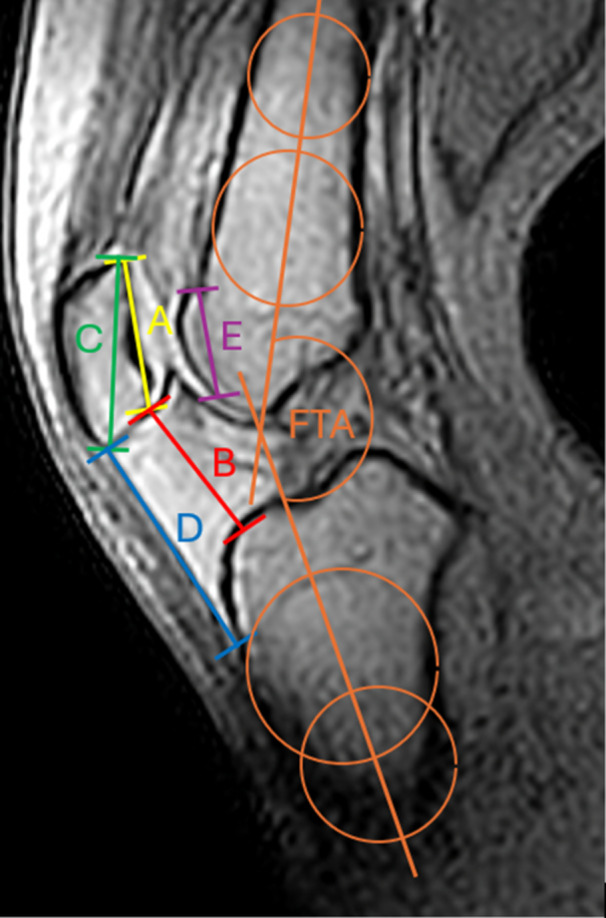

**Abbreviations**
CDI: Caton‐Deschamps‐Index
CI: confidence intervals
CT: computer tomography
dTTO: distalizing tibial tubercle osteotomy
FTA: femorotibial flexion angle
ICC: inter‐/intraclass correlation coefficient
ISR: Insall–Salvati‐Ratio
LSD: least significant difference
MRI: magnetic resonance imaging
PA: patella alta
PF: patellofemoral
PFJ: patellofemoral joint
PTI: patellotrochlear Index
ROM: range‐of‐motion
SD: standard deviations
TKA: total knee arthroplasty
TT‐TG: tibial tubercle and the trochlear groove

## INTRODUCTION

Patella alta (PA) is one of the most important factors influencing the patellofemoral joint (PFJ), leading to a 4.3‐fold increased risk for recurring patellar dislocation and a 21‐fold increased risk of developing focal patellofemoral (PF) cartilage lesions [2, 8, 14]. Beginning at borderline manifestations of PA (Insall–Salvati ratio [ISR] 1.29), several studies have reported reduced PF contact areas and increased retropatellar stress levels [26]. At the same time, some clinical studies have suggested that mild cases of PA (Caton–Deschamps index [CDI] < 1.4) may not require specific treatment, such as a distalizing tibial tubercle osteotomy (dTTO) [3, 12, 19, 22]. While these concepts may initially seem to be contradictory, the underlying reason for these conflicting findings may lie in the uncertainty surrounding the definition and diagnosis of PA. In this regard, achieving consensus on the proposed threshold values of radiologic parameters used to define and diagnose PA remains challenging [3, 8, 24]. Measurements vary depending on the imaging modality used (magnetic resonance imaging [MRI] or radiography), and the reliability of some parameters is only moderate [25, 27]. Furthermore, the influence of knee position on patellar height remains unclear. Some studies have reported that the sagittal patellar position is affected by the knee flexion angle, whereas others have found no association at low flexion angles [6, 15, 18, 25]. Consequently, uncertainty persists regarding the diagnosis of PA and the choice of treatment, as mild changes in patellar height measurements may shift a patient across commonly used thresholds for dTTO. The purpose of this study was therefore to investigate the direct influence of knee position on patellar height measurements during active muscle movement using real‐time dynamic MRI. We hypothesized that patellar height is directly associated with knee joint position during active open‐chain knee motion at low flexion angles.

## METHODS

### Study design

The present study was conducted as a diagnostic study.

### Ethical approval

The study protocol was approved by the Ethics Committee of the Medical Chamber Hamburg (ID PV7101) and was conducted in accordance with Good Clinical Practice guidelines and the principles of the Declaration of Helsinki. Written informed consent was obtained from all participants prior to data acquisition.

### Study population

The study population comprised healthy volunteers aged 18 years or older with no history of knee‐related health disorders who provided informed consent to participate in the study.

Exclusion criteria were prior episodes of instability, such as patellar dislocation or subluxation, anterior knee pain, knee trauma, prior knee surgery and limited range of motion. When feasible, both knees of one individual were analyzed depending on scanner availability.

Prior to MRI acquisition, all knees underwent clinical evaluation to confirm the absence of clinical signs of patellar maltracking, such as the J‐sign.

### Imaging and examination setup

All MR images were acquired using a 3‐Tesla (3T) MRI scanner (Ingenia, Philips). Participants were positioned supine, and a standard imaging protocol was performed, including a fat‐saturated proton density‐weighted fast spin echo (PDw FSE) sequence in the coronal, sagittal and axial planes, as well as a T1‐weighted FSE sequence in the coronal plane. Imaging was performed using a 16‐channel knee coil (Philips). The dynamic MR examination was performed using a previously validated study protocol [10]. Participants were instructed to perform an active open‐chain motion sequence, extending and flexing the knees twice consecutively within a predefined range of motion from 40° of flexion to full extension over a period of 30 s while real‐time cine MRI sequences were acquired.

### Analysis and peasurement of patellar tracking

**Figure 1:**
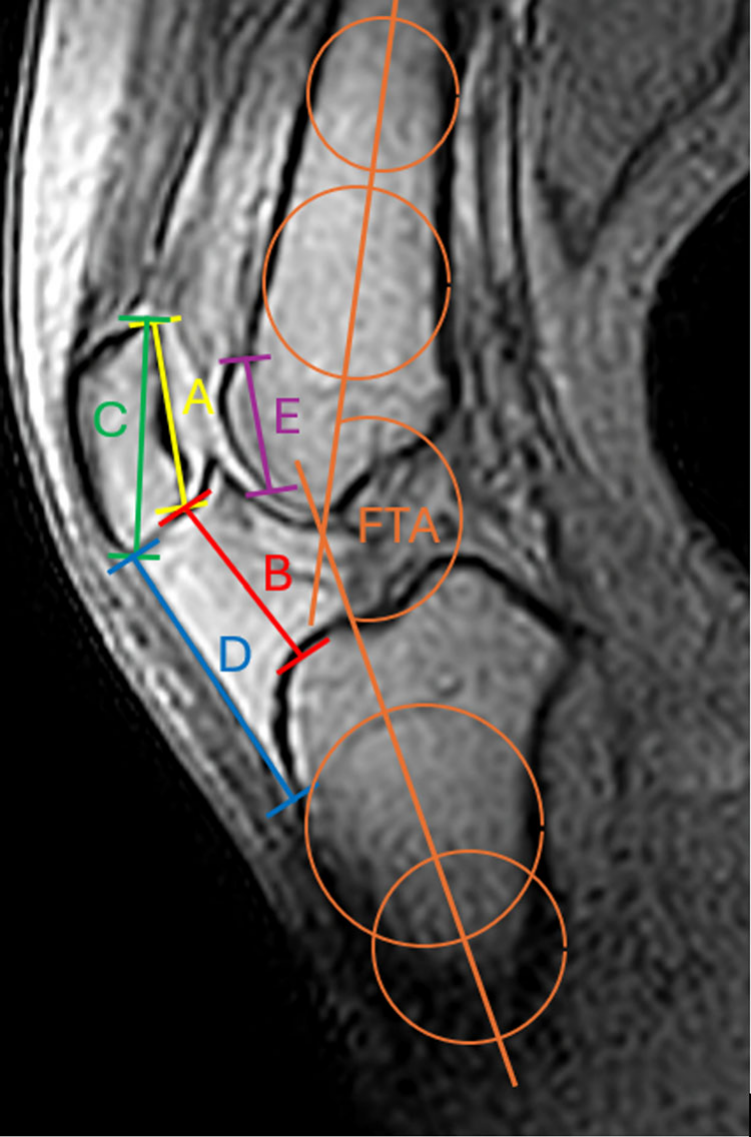
Dynamic magnetic resonance (MR) images of a right knee in sagittal orientation. The femorotibial flexion angle (FTA) (27°) was measured between the anatomic femoral and tibial axes. Patella height was determined using the Caton–Deschamps index (CDI) (B/A), the Insall–Salvati ratio (ISR) (D/C) and the patellotrochlear index (PTI) (E/A). A: Length of the articular surface of the patella, B: distance between the distal end of the retropatellar cartilage and the anterior edge of the tibia; C: diagonal patellar length, D: length of the patellar tendon, E: length of patellotrochlear overlap.

Image analysis was performed using Sectra PACS IDS7 Version 22.1 (Sectra AB, Technikering 20, SE‐583 30 Linköping). Sagittal images demonstrating the greatest patellar length were used to measure patellar height parameters. Image analysis was conducted by two fellowship‐trained orthopaedic surgeons (O.S. and C.A.) and supervised by the senior author (J.F.). Measurements were performed independently at two separate occasions with an interval of at least 6 weeks. The knee flexion angle was determined by measuring the femorotibial knee flexion angle (FTA) between the anatomic sagittal axes of the femur and tibia [9]. Patella height was assessed using the CDI, the ISR and the PTI according to Biedert et al. [16, 23] (Figure 1).

**Figure 2:**
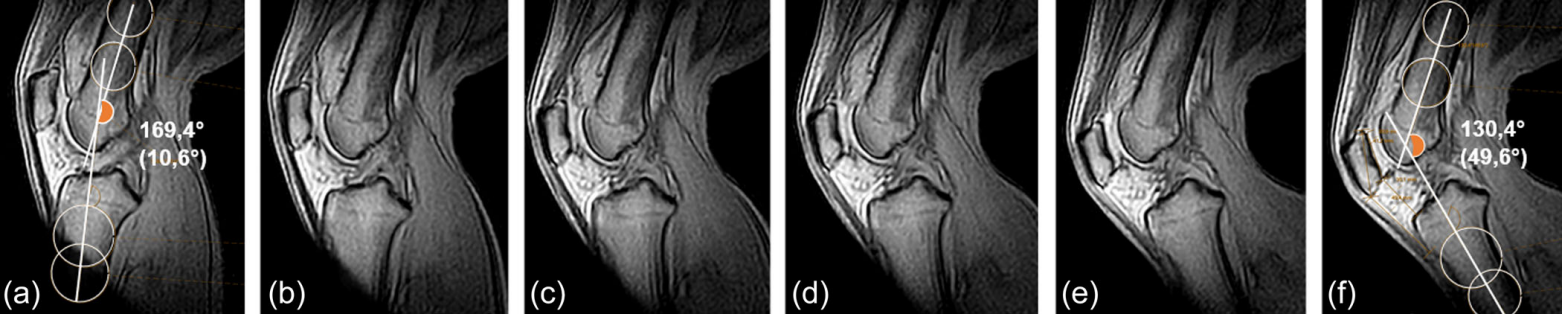
Representative image series of dynamic magnetic resonance imaging (MRI) of a knee from near extension to increasing flexion (a: 10°; b: 17°; c: 25°, d: 30°, e: 38°; f: 49° of flexion).

The measurement of all three parameters was conducted on a minimum of five distinct sagittal images per knee, with an interval of 5°–10° of flexion (Figure 2).

### Statistical analysis

Statistical analysis was performed using Microsoft Excel (version 16.31; Microsoft) and SPSS Statistics (version 29.0; IBM). A mixed‐effects model was calculated with CDI, ISR and PTI as dependent variables. Changes in PTI were analyzed using logit‐transformed values fitted with a logarithmic regression curve. Patient and knee side were included as random effects using a variance components covariance structure and modeled as repeated measures using a diagonal covariance structure. Fixed effects included the degree of flexion and knee side. Inter‐ and intraobserver agreement were assessed using the intraclass correlation coefficient (ICC) with 95% confidence intervals (CIs).

Normal distribution was evaluated using the Kolmogorov–Smirnov test. Data are presented as means with standard deviations (SD) and 95% CI. The significance level was set at *p* < 0.05.

## RESULTS

Eighty knees (45 right, 35 left) from 48 individuals (24 males, 24 females, age 26.7 ± 4.9 years, height 176 ± 9.53 cm) were included. Bilateral examinations were performed in 32 patients, whereas 16 patients contributed a single knee.

On static sagittal MRI, the mean values of all patellar height parameters were within their normal ranges (Table 1).

**Table 1:** Presentation of conventional patellar height measurements on native sagittal magnetic resonance imaging.

	**Mean**	**Standard deviation**	**Inter‐/intraclass correlation coefficient**
Flexion angle	14.13	4.29	0.76
Caton–Deschamps index	1.05	0.13	0.84
Insall–Salvati ratio	1.14	0.12	0.91
Patellotrochlear index	0.64	0.14	0.98

Within a mean active ROM from 4.3° ± 7.8° to 38.5° ± 10.4°, patellar height parameters changed significantly depending on the degree of knee flexion and the specific parameters analyzed.

**Figure 3:**
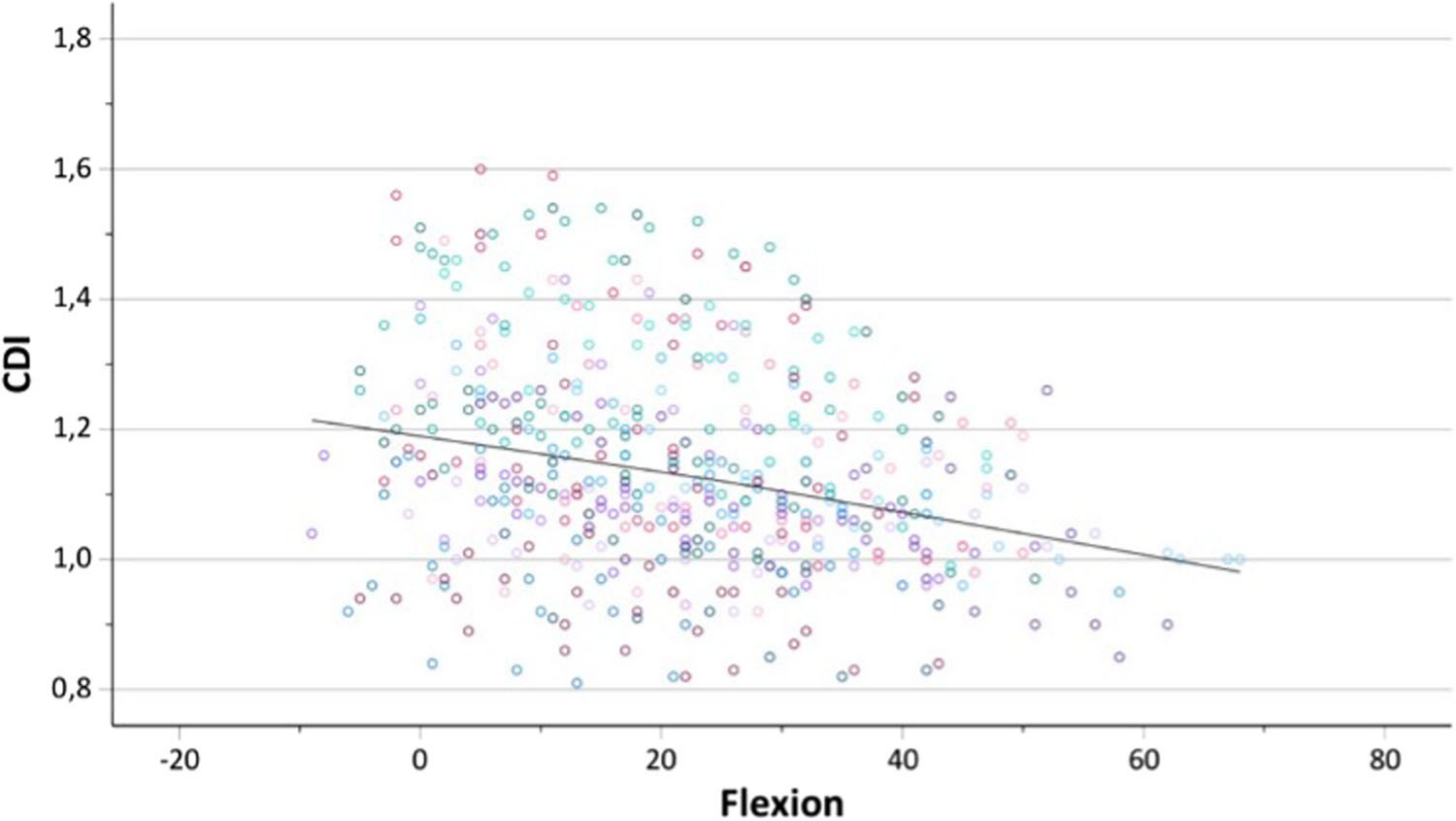
Scatter plot showing the change in the Caton–Deschamps index (CDI) as a function of increasing knee flexion.

**Figure 4:**
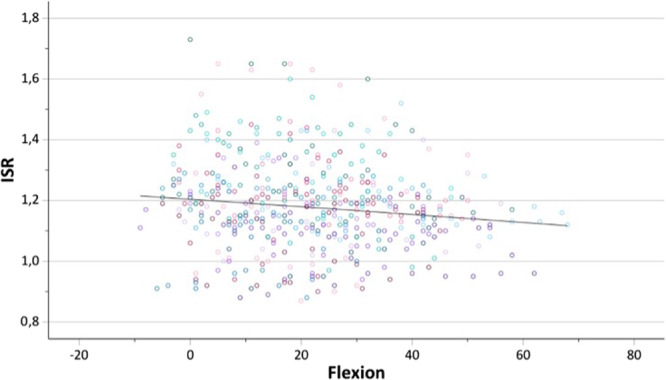
Scatter plot showing the change in the Insall–Salvati ratio (ISR) as a function of increasing knee flexion.

With each degree of increasing knee flexion, the CDI (mean in extension 1.19 ± 0.16; 95% CI: 1.10–1.19) decreased by 0.004 (*p* < 0.001) (Figure 3). In contrast, changes of the ISR (mean in extension: 1.19 ± 0.16; 95% CI: 1.15–1.24) were also significant but considerably smaller, decreasing by 0.001 (*p* < 0.001) per degree of flexion (Figure 4). For the CDI, inter‐ and intraobserver agreement were excellent (ICC = 0.91, 95% CI: 0.88–0.93) and good (ICC = 0.86, 95% CI: 0.78–0.92), respectively. For the ISR, both inter‐ and intraobserver agreement were good (ICC = 0.76; 95% CI: 0.68–0.81) and (ICC = 0.75; 95% CI: 0.59–0.85).

**Figure 5:**
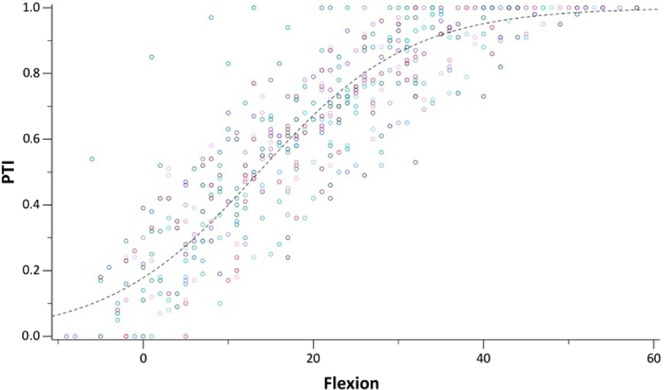
Scatter plot showing the change in the patellotrochlear index (PTI) as a function of increasing knee flexion using logistic regression analysis.

For the PTI (mean in extension 0.29 ± 0.2), strong position‐specific changes were observed (Figure 5). Inter‐ and intraobserver reliability for the PTI were excellent (ICC = 0.97; 95% CI: 0.97–0.98) and (ICC = 0.98; 95% CI: 0.97–0.99).

## DISCUSSION

The key finding of this study was that patellar height changed continuously with the degree of knee flexion at low flexion angles close to extension. This effect was reflected in the most commonly used radiologic parameters derived from dynamic MRI measurements incorporating the influence of active muscle tension. These findings highlight the importance of recognizing knee joint position as a potential confounding factor when interpreting static parameters used to determine patellar height.

Radiologic measurement of patellar height is a key aspect in the diagnosis of patellofemoral disorders. It allows for an objective evaluation of sagittal patellar position and adds valuable objectivity to the clinical assessment, which is particularly subjective in cases of PA [5]. In patients with primary patellar dislocation, PA represents one of the strongest risk factors for recurrent instability [8]. However, no consensus exists regarding the definition of a therapeutic threshold or when to consider dTTO [3, 12, 21]. According to previous studies, PA is usually diagnosed when the CDI exceeds 1.2 or when the ISR is greater than 1.2–1.3 on sagittal radiographs. These definitions have been questioned by findings from clinical studies, particularly with regard to the indication for surgical intervention [3, 12]. More importantly, methodological limitations of radiological measurement results in a certain inaccuracy when assessing patellar height. When measured on MRI, the respective parameters systematically exhibit higher values compared with conventional radiographs (CDI + 0.11, ISR + 0.18), leading to a modality‐related bias [13, 16, 27]. Moreover, most parameters used to assess PA demonstrate heterogeneous reliability, even when derived from the same imaging modality [17, 23, 25].

Another important factor in this context is the knee joint position during image acquisition. This aspect has received increasing attention in patellofemoral diagnostics, not only with regard to PA [20]. Interestingly, few studies have examined the relationship between knee flexion and patellar height measurement, and their conclusions regarding the clinical relevance have been inconsistent. Based on small case series and primarily biomechanical considerations, it is generally assumed that patellar height decreases with increasing knee flexion angles. In an investigation of nine patients, considerable changes of the sagittal patellar position were observed throughout a range of motion from 0 to 160° of flexion [11]. Another cadaveric CT‐based study demonstrated changes of patellar height with increasing knee flexion relative to the femur [6]. However, these studies were performed without active muscle tension and evaluated large ranges of motion extending into deep flexion [11]. From a biomechanical perspective, however, it is particularly important to understand the influence of muscle activity on patellar position at low flexion angles (0°–30°). Recently, Song et al. conducted a radiographic weight‐bearing study of 100 patients undergoing total knee arthroplasty (TKA). Interestingly, no significant differences in several patellar height parameters were observed when measurements obtained at 0 and 30 degrees of flexion [18]. The authors concluded that within this range, knee flexion had no relevant impact on patellar height as defined by the ISR [18]. However, in the context of dynamic patellar height assessment, the findings of this study may not be fully transferrable to native knee joints. Because TKA eliminates the natural rollback mechanism present in native knees, joint biomechanics are substantially altered, which may explain the discrepancies between our findings and those of this study [7]. In summary, the relationship between knee position and patellar height remains insufficiently understood, particularly with respect to the influence of muscle activity. Furthermore, it appears essential to define which specific parameter should be used for a given clinical indication.

The asymptomatic native knees examined in this study demonstrated normal patellar height parameters on static MRI acquired at slight knee flexion. This corresponds to the typical knee position used in standard MRI protocols with conventional knee coils. However, no clear definition of this ‘standard‐position’ currently exists. In contrast, dynamic image acquisition does not involve a predefined storing position, allowing participants to perform active open‐chain movements between 0 and 40 degrees of knee flexion. This ROM was chosen because it allows evaluation of proximal patellar tracking, as the patella engages into the trochlear groove. Sagittal reevaluation of the performed ROM revealed slight excursions into hyperextension and flexion beyond 40°. Throughout this ROM, significant changes were observed in all three parameters, which contrasts with the findings of recent studies [18]. According to our findings, the CDI of a given knee could vary by as much as 0.12 when measured at 30 degrees of flexion compared with full extension. Analysis of the PTI revealed a direct association between patellar height and increasing knee flexion, consistent with the findings of Ahmad et al., who reported similar results in a retrospective static MRI study [1]. In the preoperative evaluation of a patient, changes of this magnitude may be clinically relevant and could determine whether findings are interpreted as normal and pathological, particularly when using the PTI or CDI. In contrast, changes in the ISR during knee flexion were minimal and, despite reaching statistical significance, were of questionable clinical relevance. These results are more closely aligned with the findings of Song et al. and may suggest high reliability of the ISR in patellar height assessment [18]. However, with regard to the flexion‐dependent changes of the CDI and PTI, these findings are not surprising. The ISR describes the relationship between patellar tendon length and diagonal patellar length, both which are static and remain largely unaffected by knee flexion. Variations in bony patellar morphology or ‘relative patella alta’ caused by a short trochlear groove are therefore not considered when using the ISR, although both conditions may influence PFJ kinematics. Consequently, although the ISR may detect PA by definition, it does not account for effective patellofemoral articulation, particularly in the presence of patellofemoral pathology. In contrast, the PTI accounts for most of these factors by objectively measuring the overlap of the patellotrochlear joint surfaces. This makes the PTI a more sensitive parameter for assessing patellofemoral articulation, particularly in the evaluation of PA.

Similar considerations were reported by Becher et al., who examined changes in patellar height parameters using upright weight‐bearing MRI [4]. In their study, no influence of knee flexion on several patellar height parameters was observed, whereas only the PTI changed under axial loading in patients with PFI [4]. Narkbunnam et al. investigated the effect of weight bearing on patellar height parameters by analyzing lateral static radiographs obtained at 0°, 30° and 60° of flexion in both the supine and standing positions in a cohort of 40 healthy knees. In their study, the authors found no clinically relevant changes in patellar height with increasing knee flexion [15]. Although both studies incorporated certain dynamic aspects, image acquisition was technically performed under static conditions. In contrast, the dynamic real‐time MR images in the presented study were acquired during active open‐chain movement. Observations of patellar height at corresponding knee flexion angles were therefore made under realistic physiological muscle influence, which presents a methodological advantage.

## LIMITATIONS

This study has some limitations. First, the investigated study collective consisted exclusively of healthy knees without a history of patellofemoral disorders or knee‐related injuries, and no control group of symptomatic patients was included. In this regard, patients with PFI may exhibit altered biomechanics due to patellar maltracking. Therefore, the present findings reflect the relationship between patellar height and knee flexion in healthy knee joints and may not be fully transferrable to patients with PFI. Second, this study analyzed sagittal patellar position only, while other planes and movement directions, such as patellar tilt or rotation were not evaluated. Three‐dimensional changes of patellar position (in the coronal and transverse planes) may further improve the understanding of patellofemoral articulation in cases of PA. However, the dynamic MR imaging protocol used in this study did not allow for simultaneous three‐dimensional image acquisition, as can be achieved with computed tomography (CT). Finally, because reference values for this measuring technique are currently lacking, no a priori power analysis was performed.

## CONCLUSION

Patellar height measurements strongly depend on knee position, particularly at low flexion angles. CDI and PTI demonstrated significant changes, whereas changes of ISR were minimal. Patella height should therefore be interpreted with careful consideration of knee position and the specific index used.

## AUTHOR CONTRIBUTIONS

Oliver Swietek performed the data acquisition and drafted the manuscript. Christian Arras performed the data acquisition. Jannik Frings drafted the manuscript, supervised and conceptualized the study. Alexander Korthaus, Matthias Krause and Karl‐Heinz Frosch drafted and revised the manuscript. All authors reviewed, revised and approved the final manuscript.

## CONFLICT OF INTEREST STATEMENT

Matthias Krause and Karl‐Heinz Frosch receive consultancy fees from Arthrex (Naples, FL, USA). Jannik Frings receives consultancy fees from Conmed (Largo, FL, USA). Oliver Swietek was a research fellow, which was sponsored by the German Knee Society and Arthrex (Naplex, FL, USA). The remaining authors declare no conflicts of interest.

## ETHICS STATEMENT

The study design was approved by the local ethics committee (ID PV7101). Written informed consent was acquired from all participants.

## Data Availability

Research data are not shared.
